# Epidemiologic and Survival Analyses of Lung Cancer in Nagano Prefecture: Impact of Serial Low-Dose Computed Tomography Screening

**DOI:** 10.1016/j.jtocrr.2025.100893

**Published:** 2025-08-22

**Authors:** Tomonobu Koizumi, Kengo Otsuki, Yuichiro Maruyama

**Affiliations:** a Shinshu Cancer Center, Shinshu University Hospital, Asahi Matsumoto, Nagano, Japan; b Department of Medicine, Nagano Prefectural Kiso Hospital, Fukushima, Kiso, Nagano, Japan; c Department of Radiology, Asama Nanroku Komoro Medical Center, Komoro, Japan

**Keywords:** Population-based screening, CT screening, Mortality, Morbidity, Population-based cancer registry

## Abstract

**Introduction:**

Low-dose computed tomography (LDCT) screening for lung cancer has been implemented in many municipalities in Nagano prefecture. We analyzed epidemiologic and survival data of patients with lung cancer among municipalities in Nagano prefecture to evaluate the impact of LDCT.

**Methods:**

This was a retrospective cohort study. Data regarding the number of LDCT screenings, population, and lung cancer deaths in each municipality in 2011 to 2020 were collected and collated with information from the population-based cancer registry. These data were compared between municipalities that had continuously implemented LDCT (LDCT [+], n = 26) and those that had not (LDCT [−], n = 11).

**Results:**

The mean lung cancer crude mortality rate and standardized mortality ratio were significantly lower in LDCT (+) (82.2 ± 14.1 per 100,000 (*p* < 0.02) and 69.7 ± 11.9 (*p* < 0.005), respectively) than LDCT (−) (92.0 ± 10.1 per 100,000 and 81.6 ± 11.5, respectively). In addition, the mortality rate in each municipality was significantly negative correlated with the numbers of LDCT implementation in each municipality (*r* = 0.39). With regard to the extent of lung cancer at diagnosis, the proportion of localized lung cancer was significantly higher in LDCT (+) (39.4 ± 7.4%) than in LDCT (−) (34.6 ± 7.4%), and the proportion of localized lung cancer in each municipality was positively correlated with the number of LDCT implementation.

**Conclusion:**

This retrospective analysis in Nagano prefecture indicated that serial LDCT could contribute to a decrease in lung cancer mortality in the general population.

## Graphical abstract

**Figure:**
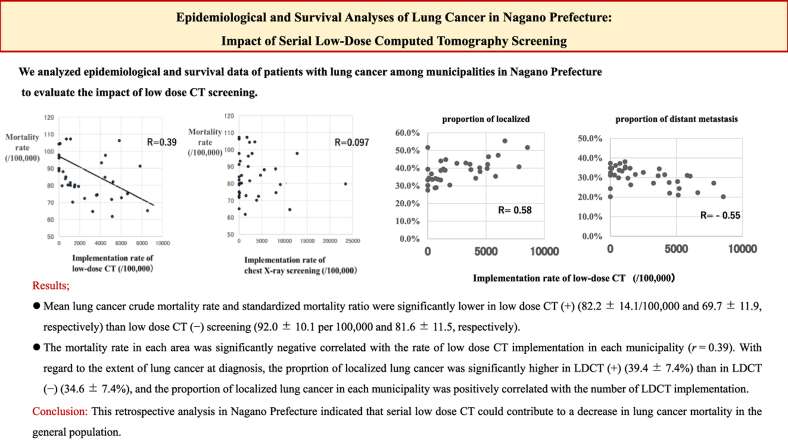

## Introduction

Lung cancer is the most common type of malignant disease and the leading cause of cancer-related death worldwide, including Japan.^1^^,^^2^ Reducing the burden of lung cancer will require a systematic and equitable implementation of evidence-based preventive strategies. Early detection and screening for lung cancer are essential for improving survival and reducing the mortality rate.^3^, ^4^, ^5^

There have been many reports that chest low-dose computed tomography (LDCT) can detect more of these lesions at an earlier stage than chest radiography (X-ray).^5^, ^6^, ^7^ Two large randomized controlled trials designed to evaluate LDCT screening for lung cancer, the National Lung Screening Trial (NLST) in the United States^8^ and Nederlands-Leuvens Longkanker Screenings Onderzoek (NELSON) in Europe,^9^ significantly reduced lung cancer mortality in patients with heavy smokers compared with chest X-ray screening and usual care, respectively. Subsequently, a recent meta-analysis of nine clinical studies found that LDCT screening significantly reduced lung cancer mortality, although this did not affect overall mortality in the studies.^10^ Thus, the survival benefit remained to be limited and the risk of overdiagnosis was acknowledged; however, LDCT screening was prioritized because of its proven sensitivity and ability to detect early-stage lung cancer.

The Nagano LDCT group initially began LDCT screening using a mobile CT unit,^7^^,^^11^^,^^12^ and exhibited the usefulness of this method. For example, we found better lung cancer survival among never-smokers in the LDCT screening group than the chest X-ray screening group.^12^ LDCT screening for lung cancer has now been extended in many municipalities in Nagano prefecture, resulted that 61 of the 77 municipalities in Nagano prefecture had implemented LDCT in 2017.

In the present study, we evaluated the epidemiologic and survival data of patients with lung cancer among municipalities in Nagano prefecture to see the impact of LDCT screening. We also evaluated the implementation status of LDCT screening in each municipality and performed a comparison between municipalities that had and had not yet implemented LDCT.

## Methods

LDCT screening in Nagano prefecture has been performed by each municipality initiative. Each municipality has contacted health screening organizing institutes having a LDCT mobile unit. The entry criterion of age 40 years or older was applied consistently in every municipality, but sex, smoking history, and screening interval of participants were not regulated in each municipality. In addition, the annual schedule and continuity of LDCT screening were also dependent on each municipality. Annual numbers of LDCT screenings were provided by the health screening organizations: Nagano Health Promotion Corporation, Matsumoto City Medical Association, and JA Nagano Kouseiren Health Control Center of Saku Central Hospital. The municipalities were divided into the LDCT (+) (n = 26) that had implemented LDCT screening over the study period (2011–2020) and the LDCT (−) (n = 11) that had not implemented or continued LDCT screening. We excluded 35 villages because of their small populations and five towns because of insufficient data on LDCT implementation status.

Data on the number of chest X-ray screenings, population, and lung cancer deaths from 2011 to 2020 were collected for this retrospective cohort study. These data were obtained from the Portal Site of Official Statistics of Japan (e-Stat), the official government statistics portal.^13^ The analysis focused on individuals aged 40 years and older, as eligibility for LDCT screening in each municipality was limited to this age group. For each year and municipality, the lung cancer crude mortality rate was calculated as the number of lung cancer deaths divided by the population. The standardized mortality ratio (SMR) was calculated by stratum using the Japanese Mortality Database operated by the National Institute of Population and Social Security Research.^14^ SMR was determined by dividing the number of observed deaths by the number of expected deaths, calculated using age-specific death rates for lung cancer in Japan. These rates were derived by dividing the number of deaths by the population of Nagano prefecture, and by individual municipality for each year. The national SMR was given a reference value of 100 for use as a reference in normalization.

Data from the population-based cancer registry was used with permission from Nagano prefecture. Using information from 2011 to 2020, we evaluated the number of lung cancer cases, detection methods, and extent of disease for each year and municipality. The annual incidence rate of newly diagnosed lung cancer was calculated for each municipality, and the age-adjusted incidence rate was calculated over the entire study period using the 1985 model Japanese population as the standard. Incidence rates were also calculated specifically for individuals aged 40 years and older. On the basis of the population-based cancer registry, we divided the detection methods of lung cancer into screening, incidental, and others in the present study. Detection of screening included population- and individual-based health screening. Detection of others included symptomatic, unknown, and missing cases. In addition, the population-based cancer registry classified extent of disease into four categories—localized, regional lymph node metastasis, adjacent organ invasion, and distant metastasis, corresponding to the Union for International Cancer Control and the American Joint Committee on Cancer TNM classification version 8^15^—T1-2N0M0, T1N1-2M0, T3-4N0-2M0, and TanyN3M0 or TanyNanyM1, respectively. These epidemiologic and survival data were then compared between LDCT (+) and LDCT (−).

The study was approved by the Institutional Review Board of Shinshu University School of Medicine (approval number 6161) and was conducted in accordance with the principles of the Declaration of Helsinki. The need for written informed consent for each subject was waived by the Shinshu University School of Medicine Biological and Medical Research Ethics Committee because of the retrospective nature of the study and handling with completely anonymized data.

## Statistical Analysis

The results are presented as the mean (± SD). Pearson’s correlation coefficient and corresponding *p* values are presented for assessment of correlations. Differences between LDCT (+) and LDCT (−) were compared using the two-tailed Student’s *t* test. Statistical analyses were performed using EZR Statistics (Saitama Medical Center, Jichi Medical University, Saitama, Japan).^16^ Trend analyses for mortality and age-adjusted incidence rates were conducted using the Joinpoint regression model. A piecewise log-linear regression model was applied using Joinpoint version 5.3.0 (National Cancer Institute; https://surveillance.cancer.gov/joinpoint/). The direction and magnitude of trends were evaluated using the annual percentage change (APC). In all analyses, *p* less than 0.05 was taken to indicate statistical significance.

## Results

The 37 municipalities in Nagano prefecture were divided into two groups: the LDCT (+) (n = 26) and the LDCT (−) (n = 11). The LDCT (+) included 15 cities (median population, 34,230; range, 18,938–142,841) and 11 towns (median population, 6795; range, 3098–15,244). Six municipalities have continued LDCT screening since before 2000 years, the rest of the municipalities started LDCT screening before 2010 and have continued the screening. The median number of LDCT implementation in LDCT (+) per each municipality was 3475/100,000 population ranging from 309 to 8543/100,000 during the study period. The LDCT (−) group included four cities (median population, 41,163; range, 14,331–230,243) and seven towns (median population, 7744; range, 3566–13,687).

**Figure 1:**
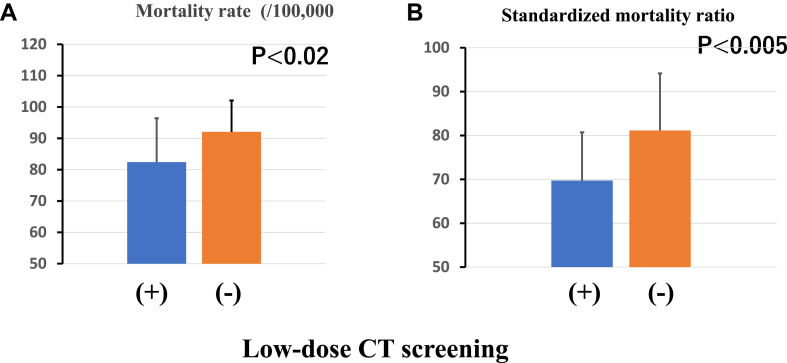
Comparison of the mean lung cancer crude mortality rate (*A*) and standardized mortality ratio (*B*) between areas with and without low-dose CT screening. CT, computed tomography.

**Figure 2:**
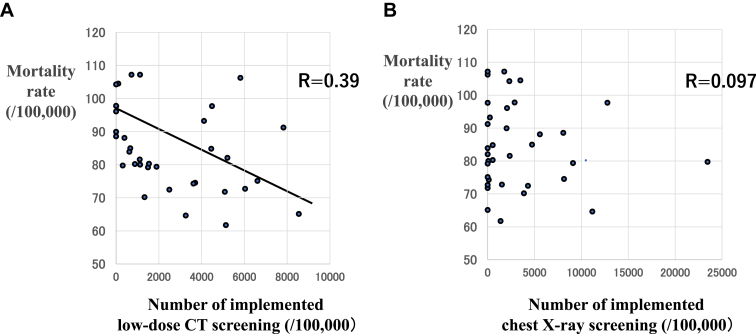
Correlation between crude lung cancer mortality rate and the number of implemented low-dose CT screening (*A*) and chest X-ray screening (*B*). CT, computed tomography; X-ray, chest rdiography.

The mean lung cancer crude mortality rate in LDCT (+) was 82.2 ± 14.1/100,000 population, which was significantly lower than the value in LDCT (−) of 92.0 ± 10.1/100,000 population (*p* < 0.02) (Fig. 1*A*). SMR in Nagano prefecture was low compared with the national value of 100. In addition, the SMR was significantly lower in LDCT (+) than LDCT (−) (69.7 ± 11.9 versus 81.6 ± 11.5, respectively; *p* <0.005) (Fig. 1*B*). The mean lung cancer crude mortality rates in each municipality were significantly negatively correlated with the numbers of LDCT implementation (*r* = 0.39) (Fig. 2*A*). However, there was no significant correlation between mean lung cancer crude mortality rate and X-ray screening implementation rate (Fig. 2*B*).

**Figure 3:**
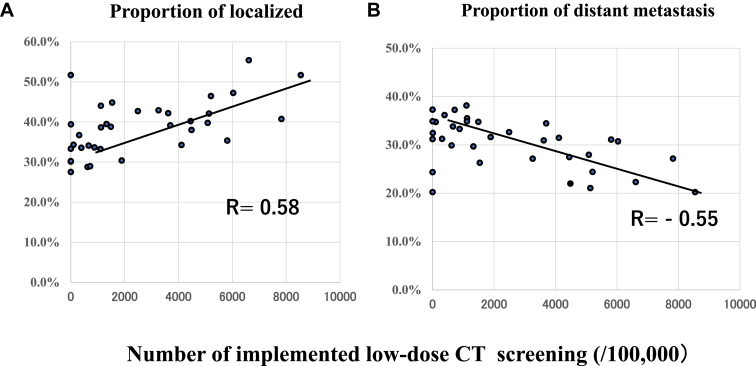
(*A*) Correlation between the frequency of localized staged lung cancer and the number of implemented low-dose CT screening. (*B*) Correlation between the frequency of distant metastasis–stage lung cancer and the number of implemented low-dose CT screening. CT, computed tomography.

**Figure 4:**
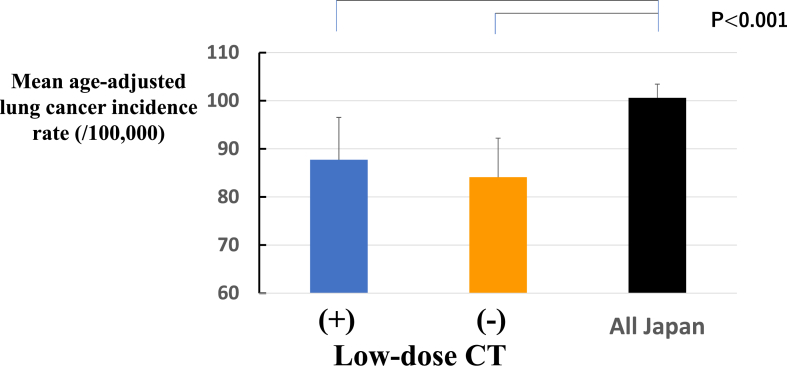
Comparison of mean age-adjusted lung cancer incidence rate between areas with and without low-dose CT in Nagano prefecture and all Japan. CT, computed tomography.

**Figure 5:**
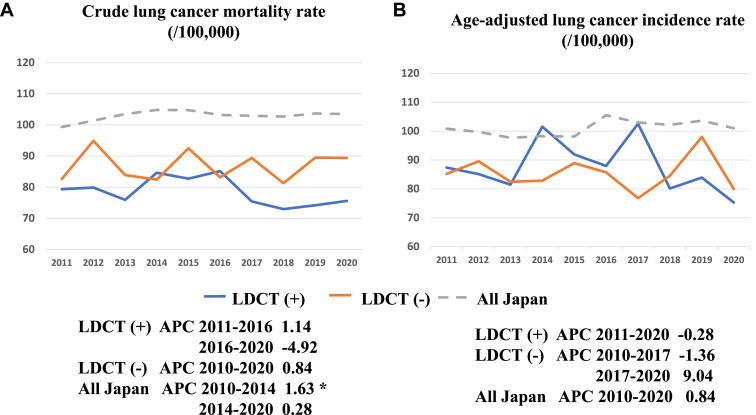
Trends of crude lung cancer mortality rate (*A*) and age-adjusted lung cancer incidence rate (*B*) in LDCT (+), LDCT (−) and all Japan from 2011 to 2020. ∗APC was considered statistically significant (*p* < 0.05). APC, annual percentage change; LDCT, low-dose computed tomography.

The methods of lung cancer detection and the extent of lung cancer diagnosis in the population-based cancer registry are summarized in Table 1. The proportion of screening-detected lung cancer was significantly higher in LDCT (+) than in LDCT (−). In contrast, the proportion of others was significantly lower in LDCT (+) than in LDCT (−), although others included missing data of 1.2 % in LDCT (+) and 1.7 % in LDCT (-), respectively. The proportion of diagnosed as localized lung cancer was significantly higher in LDCT (+) than LDCT (−), and the proportion in each municipality was positively correlated with the number of LDCT implementation (Fig. 3*A*). There was no significant difference in the frequency of diagnosed as distant metastasis between LDCT (+) and LDCT (-) (Table 1), but a negative correlation was detected between proportion of distant metastasis in each municipality and the numbers of LDCT implementation (Fig. 3*B*). Mean age-adjusted lung cancer incidence rates are plotted in Figure 4. The mean incidence rate in LDCT (+) during study period was 87.7±8.8/100,000 population and 84.1±8.1/100,000 population, respectively. There were no significant differences between LDCT (+) and LDCT (-), but these mean incidence rates were both significantly lower than the national average (100.6 ± 2.8/100,000 population). Figure 5 illustrates the trends in lung cancer mortality and incidence rates during the study period. Although mortality tended to increase in both the nationwide data and the LDCT (−), a declining trend was seen in the LDCT (+) in the later years (APC −4.92% from 2016 to 2020) (Fig. 5*A*). Although the incidence rates revealed year-to-year variation in both the LDCT (+) and LDCT (−), the age-adjusted lung cancer incidence rate in the LDCT (+) exhibited a decreasing trend over the study period (APC −0.28% from 2011 to 2020) (Fig. 5*B*). In contrast, the nationwide incidence rate exhibited an increasing trend (APC 0.84% from 2011 to 2020). The trend in Nagano prefecture exhibited a slight decline in incidence (APC −0.45% from 2011 to 2020) (data not provided).

**Table 1:** The Proportion of Lung Cancer Detection (%) and Extents of Lung Cancer (%) in the Present Study

Detection Method	LDCT (+)	LDCT (-)
Screening	19.8 ± 1.0	15.0 ± 2.0^a^
Incidental	41.1 ± 4.0	40.7 ± 6.0
Others	39.1 ± 4.6	44.3 ± 5.1^b^
Extent of disease	LDCT (+)	LDCT (-)
Localized	39.4 ± 7.4	34.6 ± 7.4^b^
Regional lymph node metastasis	8.6 ± 2.5	11.1 ± 2.3
Adjacent organ invasion	7.7 ± 2.2	8.1 ± 2.7
Distant metastasis	29.4 ± 4.9	31.6 ± 5.4

*Note*: Data from the population-based cancer registry.

LDCT, low-dose computed tomography.

a *p <* 0.001.

b *p <* 0.03.

## Discussion

The present study was performed to evaluate the impact of LDCT screening using epidemiologic and survival analyses in the general population of Nagano prefecture, where LDCT has been implemented for an extended period. Although the present results were from a retrospective analysis and lacks the data of smoking history and sex differences, we found that lung cancer mortality rate was significantly lower in the LDCT (+) than the LDCT (−). Moreover, the increased number of LDCT implementation per population was positively correlated with decreased lung cancer mortality, whereas no such association was observed with the numbers of chest X-ray screening.

Previously, we reported a high detection rate of early-stage lung cancer in Nagano prefecture compared with the national average, using population- and hospital-based cancer registry data from 2012 to 2014.^17^ Although other health screening might be related to the significantly higher proportion of localized lung cancer in the present study, our data suggested that LDCT screening contributed to the high early detection. Furthermore, our data revealed a lower lung cancer mortality in the LDCT (+) and a significant correlation between the numbers of LDCT implementation and reduced mortality. Notably, Nagano prefecture has consistently maintained the lowest age-adjusted lung cancer mortality rate in Japan.^18^ Although the decline in lung cancer mortality in the LDCT (+) was not statistically significant, it contrasted with the steady increase observed in the LDCT (−). Taken together, these findings suggest that continuous LDCT screening may contribute to reduced lung cancer mortality.

The population-based cancer registry in Nagano prefecture was established in 2010, with a death certificate–only rate of 28.8% in its first year. However, the death certificate–only rate decreased significantly to 6.0% in 2011 and has remained below 5.0% since 2014 (https://www.pref.nagano.lg.jp/shippei-kansen/gan/touroku/documents/gantourokuhoukokusyo2020.pdf). Therefore, the Nagano Prefectural Cancer Registry maintained high data quality throughout the study period, and we believe that our epidemiologic data on lung cancer reliably reflect clinical practice in each municipality.

We were unable to evaluate the impact of smoking on our results because of insufficient information on smoking history across the entire study period. Although the positive findings of the NLST and NELSON trials established the value of LDCT screening in heavy smokers,^8^^,^^9^ limited evidence supports its efficacy in non- or light smokers.^8^^,^^9^ A retrospective cohort study performed in Korea reported a high detection rate of lung cancer by LDCT in never-smokers, with 92.7% of cases detected at an early-stage and associated with survival benefit.^19^ Similarly, Nawa et al.^20^ reported a reduction in lung cancer mortality through LDCT screening in a population that included non- and light smokers in the Hitachi city (Ibaraki Prefecture), Japan. Our previous Nagano LDCT study also reported better lung cancer survival in never-smokers screened by LDCT in comparison with those screened by chest X-ray.^12^ Given the increasing importance of addressing lung cancer in non- and light smokers, a randomized controlled trial of LDCT screening in this population (the JECS study; The Japanese Randomized Trial for Evaluating the Efficacy of Low-dose Thoracic CT Screening for Lung Cancer) is currently underway in Japan.^21^ The results are expected to provide valuable information for lung cancer prevention.

Furthermore, we were unable to analyze sex differences in lung cancer mortality in the present study because of the lack of sex-specific mortality data for each area. The proportion of lung cancer cases among never-smokers and women is higher in Korea, Japan, and People's Republic of China than in Europe and the United States.^3^^,^^22^ *EGFR*-mutated adenocarcinoma, which is more common in women and never-smokers, may contribute to these higher prevalence rates in these countries.^23^ In addition, other countries have reported increasing trends in lung cancer among never-smokers.^24^ These observations highlight the importance of developing lung cancer prevention strategies for the general population. Our findings suggest that LDCT screening could play a valuable role in such strategies, particularly in Japan, and support evaluation of the efficacy of LDCT screening in the general population and not just in high-risk groups.

With regard to age-adjusted lung cancer incidence rates, recent national data revealed a decreasing trend across the general population, including Nagano prefecture.^2^ In the present study, we found a slight increase in the age-adjusted lung cancer incidence in individuals aged 40 years and older over the study period. However, the overall lung cancer incidence in Nagano prefecture remained lower than the national average and exhibited a declining trend. Several studies reported that the introduction of LDCT screening can initially increase the incidence of lung cancer because of early detection.^9^^,^^10^^,^^19^^,^^25^ LDCT screening began in Nagano prefecture in the 1990s, and has been maintained for a long period. We speculated that this long-term implementation may have been associated with reduced lung cancer morbidity. Nevertheless, many factors influence lung cancer incidence, including smoking, asbestos exposure, air pollution, and aging, so the direct impact of long-term LDCT screening on incidence remains unclear. We believed that epidemiologic findings from Nagano prefecture could provide valuable insights for lung cancer prevention. Further research will be necessary to clarify the long-term effects of LDCT screening on the incidence of lung cancer.

The present study had several limitations. First, we were unable to calculate age-adjusted mortality rates and the sex differences for each municipality, which limited our ability to accurately evaluate area-specific mortality rates. Second, LDCT screening was not conducted in a randomized manner. Although the entry criterion of age 40 years and older was applied consistently across municipalities, other factors, such as upper age limits, smoking history, baseline comorbidities, and screening intervals, were not standardized. Third, we lacked data on the interval between initial screening and lung cancer diagnosis because of the retrospective nature of the study. To enhance the accuracy and comparability of lung cancer screening outcomes, future studies should aim to unify recruitment criteria and LDCT screening intervals across municipalities. Despite these limitations, our findings provide valuable insights into the effectiveness of LDCT-based lung cancer screening, particularly for populations in Japan and East Asia. The results support the utility of LDCT as a population-based screening strategy for lung cancer.

In summary, this retrospective analysis in Nagano prefecture, Japan, revealed that serial LDCT screening may contribute to reduced lung cancer mortality in the general population.

## CRediT Authorship Contribution Statement

**Tomonobu Koizumi:** Conceptualization, Methodology, Investigation, Writing - review & editing.

**Kengo Otsuki:** Methodology, Investigation, Writing - review & editing.

**Yuichiro Maruyama:** Conceptualization, Methodology, Investigation, Writing - review & editing.

## Disclosure

Dr. Koizumi reports receiving honoraria from AstraZeneca, Novartis Pharma, and Daiichi Sankyo Healthcare. The remaining authors declare no conflict of interest.
